# Functional distribution of Ca^2+^-coupled P2 purinergic receptors among adrenergic and noradrenergic bovine adrenal chromaffin cells

**DOI:** 10.1186/1471-2202-8-39

**Published:** 2007-06-14

**Authors:** Ângelo R Tomé, Enrique Castro, Rosa M Santos, Luís M Rosário

**Affiliations:** 1 Center for Neurosciences and Cell Biology, University of Coimbra, Coimbra, Portugal; 2 Department of Biochemistry, Faculty of Sciences and Technology, University of Coimbra, P.O. Box 3126, 3001-401 Coimbra, Portugal; 3 Department of Biochemistry, Molecular Biology and Physiology, Faculty of Medicine and Health Sciences, University of Las Palmas de Gran Canaria, Las Palmas, Spain

## Abstract

**Background:**

Adrenal chromaffin cells mediate acute responses to stress through the release of epinephrine. Chromaffin cell function is regulated by several receptors, present both in adrenergic (AD) and noradrenergic (NA) cells. Extracellular ATP exerts excitatory and inhibitory actions on chromaffin cells via ionotropic (P2X) and metabotropic (P2Y) receptors. We have taken advantage of the actions of the purinergic agonists ATP and UTP on cytosolic free Ca^2+ ^concentration ([Ca^2+^]_i_) to determine whether P2X and P2Y receptors might be asymmetrically distributed among AD and NA chromaffin cells.

**Results:**

The [Ca^2+^]_i _and the [Na^+^]_i _were recorded from immunolabeled bovine chromaffin cells by single-cell fluorescence imaging. Among the ATP-sensitive cells ~40% did not yield [Ca^2+^]_i _responses to ATP in the absence of extracellular Ca^2+ ^(Ca^2+^_o_), indicating that they expressed P2X receptors and did not express Ca^2+^- mobilizing P2Y receptors; the remainder expressed Ca^2+^-mobilizing P2Y receptors. Relative to AD-cells approximately twice as many NA-cells expressed P2X receptors while not expressing Ca^2+^- mobilizing P2Y receptors, as indicated by the proportion of cells lacking [Ca^2+^]_i _responses and exhibiting [Na^+^]_i _responses to ATP in the absence and presence of Ca^2+^_o_, respectively. The density of P2X receptors in NA-cells appeared to be 30–50% larger, as suggested by comparing the average size of the [Na^+^]_i _and [Ca^2+^]_i _responses to ATP. Conversely, approximately twice as many AD-cells expressed Ca^2+^-mobilizing P2Y receptors, and they appeared to exhibit a higher (~20%) receptor density. UTP raised the [Ca^2+^]_i _in a fraction of the cells and did not raise the [Na^+^]_i _in any of the cells tested, confirming its specificity as a P2Y agonist. The cell density of UTP-sensitive P2Y receptors did not appear to vary among AD- and NA-cells.

**Conclusion:**

Although neither of the major purinoceptor types can be ascribed to a particular cell phenotype, P2X and Ca^2+^-mobilizing P2Y receptors are preferentially located to noradrenergic and adrenergic chromaffin cells, respectively. ATP might, in addition to an UTP-sensitive P2Y receptor, activate an UTP-insensitive P2Y receptor subtype. A model for a short-loop feedback interaction is presented whereby locally released ATP acts upon P2Y receptors in adrenergic cells, inhibiting Ca^2+ ^influx and contributing to terminate evoked epinephrine secretion.

## Background

Adrenal chromaffin cells secrete norepinephrine and the stress-related hormone epinephrine in response to acetylcholine output from splanchnic nerve terminals. Its function is modulated by other transmitters and mediators released from either nerve terminals, adjacent cells or the cells themselves (for review see [1]). Among these regulators is ATP, which is co-released with transmitters and catecholamines since it is present in large amounts in secretory vesicles [2,3]. It has long been known that extracellular ATP exerts multiple regulatory actions on catecholamine secretion from either whole adrenal glands or isolated chromaffin cells. Indeed, ATP evokes secretion in a Ca^2+^-dependent manner [4-8]. There are also reports showing that ATP and other ATP receptor agonists inhibit voltage-sensitive Ca^2+ ^channels (via G_i_/G_o _proteins) and inhibit depolarization-evoked catecholamine release [4,9-14]. These channels are an essential component of the stimulus-secretion coupling cascade in chromaffin cells [15].

Historically, recognition of the two major chromaffin cell phenotypes (epinephrine-secreting or adrenergic, hereby referred to as AD-cells, and norepinephrine-secreting or noradrenergic, NA-cells) was based on morphological differences, secretory specificity of purified preparations and, later, on immunocytochemical labeling involving the use of antibodies against enzymes related to catecholamine biosynthesis [16]. AD- and NA-cells appear to be differentially regulated by various transmitters or mediators (e.g. NO, histamine, angiotensin II and opioid peptides), and there is immunocytochemical and other evidence that some of its receptors are differentially distributed among both cell subtypes [17-22]. We have also provided functional and pharmacological evidence that chromaffin cell subpopulations express distinct ATP receptor subtypes, i.e. uridine 5'-triphosphate (UTP)-sensitive metabotropic receptors and suramin-blockable ionotropic receptors coupled to Ca^2+ ^influx [7,23]. It remains however unknown whether specific purinoceptor subtypes are asymmetrically distributed among AD- and NA-cells. This is an important issue, since it may shed light on the mechanisms regulating acute stress responses in superior organisms.

P2X receptors are Ca^2+^-permeable and provide an important Ca^2+ ^influx pathway, both in neurons and other cell types (for review see [24]). The metabotropic (P2Y) purinoceptors are classical 7-transmembrane domain receptors coupled to either G_q/11 _or G_i/o _proteins and, predominantly, to Ca^2+ ^release from intracellular stores, with at least eight known subtypes (for review see [24-26]).

Expression of P2Y_2 _and P2Y_12 _(formerly known as P2Y_ADP _or P_2T_) purinoceptors in rat chromaffin cells was suggested by immunocytochemistry and [^35^S]GTPγS autoradiography studies on adrenal medulla sections [27,28]. Ennion *et al. *[13] demonstrated the presence of G_i/o_-linked, adenine nucleotide-specific P2Y_12 _receptors in bovine chromaffin cells; in addition, the authors suggested the presence of an as yet unidentified UTP-sensitive, G_i/o_-coupled P2Y receptor. Activation of both receptor subtypes inhibits voltage-sensitive Ca^2+ ^channels and exocytosis [10,13]. In contrast to the putative UTP-sensitive receptor, P2Y_12 _receptors in chromaffin cells are seemingly uncoupled to Ca^2+ ^release from intracellular stores [13].

We have previously shown that ATP and UTP, at saturating concentrations, evoke rises in cytosolic free Ca^2+ ^concentration ([Ca^2+^]_i_) of similar amplitude in a subpopulation of bovine chromaffin cells lacking P2X receptors [23]. These nucleotides are seemingly equipotent for the Ca^2+ ^mobilizing P2Y receptors, as suggested by [Ca^2+^]_i _studies in the absence of external Ca^2+ ^[8]. Both characteristics are consistent with UTP receptors (P2Y_2 _and/or P2Y_4 _[29]) playing a major role in the ATP responses under conditions where Ca^2+ ^does not enter cells *via *ATP-gated channels. Thus, UTP appears to be the agonist of choice for monitoring the action of Ca^2+^-mobilizing P2Y receptors in bovine chromaffin cells. In contrast, 2-methylthioadenosine 5'-triphosphate (2-MeSATP), formerly thought to behave as a specific P2Y agonist, activates selected P2X and P2Y receptor subtypes in different cell types [30]. (It is actually specific for P2X receptors in guinea-pig [31,32] and bovine chromaffin cells [33].)

In this work, we have used ATP and UTP as purinergic agonists to investigate the distribution of Ca^2+^-coupled P2X and P2Y receptors among adrenergic and noradrenergic bovine chromaffin cells by single-cell fluorescence imaging. We found that, although neither of the major purinoceptor types can be ascribed to a particular cell phenotype, P2X and Ca^2+^-mobilizing P2Y receptors are preferentially located to noradrenergic and adrenergic chromaffin cells, respectively.

## Results

### Identification of chromaffin cell phenotypes

**Figure 1:**
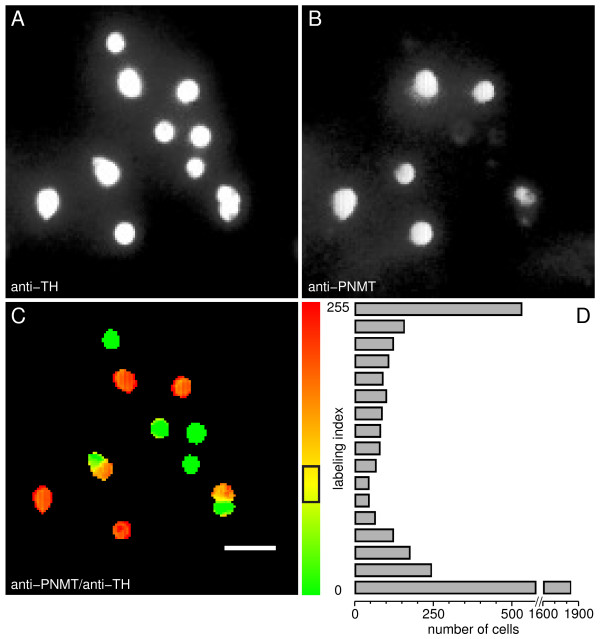
**Immunocytochemical identification of chromaffin cells**. **A**. Fluorescence image of cells labeled with anti-TH antibodies (total population); **B**. Immunostaining with anti-PNMT antibodies (adrenergic cells); **C**. Pseudocolor image of PNMT/TH immunofluorescence ratio (labeling index) and corresponding scale (right). Yellow corresponds to an index of ~98, the center of the mis-identification region (84–112) indicated by the black box on the scale; **D**. Frequency distribution histogram for all cells analyzed in this study (number of cells *vs*. labeling index, measured as the averaged index over the region covered by the cell). Cells with a labeling index above the upper threshold (toward red) were considered PNMT^+ ^(adrenergic) cells. Conversely, cells with an index below the lower threshold were considered TH^+^/PNMT^- ^(noradrenergic) cells. Controls without primary antibodies showed a very faint, almost indiscernible fluorescence, in photographs taken using the same exposure times. Scale bar = 50 μm.

N-phenyl ethanolamine N-methyl transferase (PNMT) is a specific enzyme for epinephrine biosynthesis; tyrosine hydroxylase (TH) is the rate-limiting enzyme for catecholamine biosynthesis. Thus, PNMT marks AD-cells and its absence in TH^+ ^cells indicates that TH^+^/PNMT^- ^cells are NA-cells. Fig. 1A and 1B depicts a typical immunocytochemical identification of chromaffin cells using anti-TH and anti-PNMT antibodies. A labeling index, scaled to the 0–255 range of an 8-bit image, was calculated bit-by-bit for each cell in a field by taking the ratio of background-corrected rhodamine fluorescence (indicative of PNMT expression) over background-corrected fluorescein fluorescence (indicative of TH expression). The labeling indices were used for the generation of pseudocolor images (Fig. 1C), thus allowing unambiguous identification of AD-cells (TH^+^/PNMT^+^; high index values, red) and NA-cells (TH^+^/PNMT^-^; low index values, green). An extra bonus of this analysis was to reveal that some cells actually gathered in small clusters (mostly 2 or 3 cells) displaying both phenotypes.

A fraction of the cells exhibited a yellowish or less marked red or green pseudocolor, suggesting a variable PNMT expression and, in some instances, raising doubts as to the identification of the respective phenotype. In order to resolve this issue, a frequency distribution histogram of the labeling index was constructed (Fig. 1D). Most of the cells fell at the extreme edges of the histogram and could, therefore, be clearly identified as either AD- or NA-cells. In order to set appropriate upper and lower threshold levels for error detection, a gamma distribution function was fitted to each side of the histogram (P = 0.005, i.e. 1 out of 200 cells mis-identified). The resulting cut-off levels are delimited by the black box in the pseudocolor scale (Fig. 1C). Cells that fell within this region were considered to be either PNMT false positives or false negatives, being therefore discarded from further analysis. Through this analysis AD-cells and NA-cells were found to account for 41% and 59% of the whole chromaffin cell population under study, respectively.

### ATP- and UTP-evoked [Ca^2+^]_i _rises in chromaffin cell subtypes

[Ca^2+^]_i _changes evoked by ATP receptor agonists were monitored by digital fluorescence imaging of the F_340_/F_380 _fura-2 fluorescence ratio (ΔR). Only cells that displayed sizeable [Ca^2+^]_i _responses to acetylcholine nicotinic receptor agonists (nicotine or 1,1-dimethyl-4-phenylpiperazinium iodide, DMPP), established functional markers of chromaffin cells [34], were considered for the study. To this end, cells were perifused at the tail of the experiments with either 10 μM nicotine or 10 μM DMPP for brief periods of time. Cells that were part of small clusters were considered for the study, provided that they could be unambiguously distinguished from each other through a combination of immunofluorescence staining and [Ca^2+^]_i _responsiveness.

**Figure 2:**
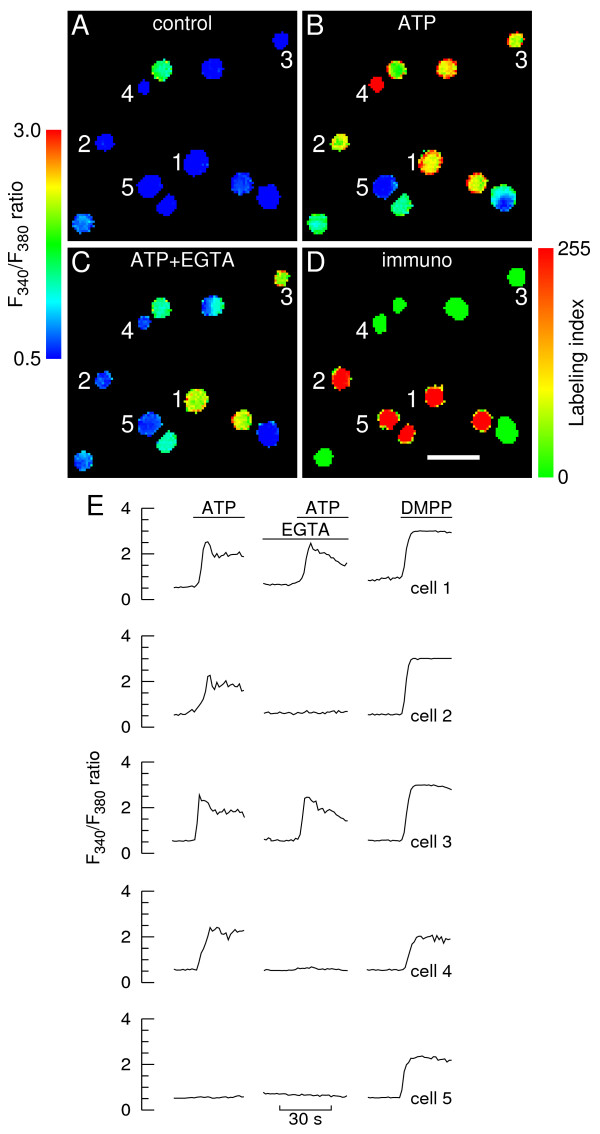
**Calcium responses to ATP in immunolabeled chromaffin cells**. **A-C**. Calcium images showing a group of chromaffin cells before (A, control), during stimulation with 100 μM ATP in presence of extracellular calcium (B) and during stimulation in the virtual absence of extracellular calcium (C, "ATP+EGTA"). At the end of each experiment cells were stimulated with 10 μM DMPP. Cells were allowed to rest for 10 min between consecutive stimulations. The fura-2 fluorescence ratio F_340_/F_380 _was determined for each cell in a field on a pixel-by-pixel basis. Images were coded in pseudocolor to show differences in the F_340_/F_380 _ratio. The images corresponding to ATP stimulation (B and C) were captured ~5 s after the ATP challenges (i.e. at the response peak in presence of extracellular calcium); **D**. Pseudocolor image of PNMT/TH immunofluorescence ratio (labeling index) and corresponding scale. Intensely colored red and green cells are adrenergic and noradrenergic cells, respectively. Scale bar = 50 μm. **E**. Time courses of changes in F_340_/F_380 _fluorescence ratio for three AD-cells (cells 1, 2 and 5, also depicted in A-D) and two NA-cells (cells 3 and 4). The lines denote superfusions with ATP or DMPP in the presence or virtual absence (middle traces) of extracellular calcium.

Typical pseudocolor fura-2 fluorescence images are depicted in Fig. 2(A–C), together with the respective immunostaining identification (labeling index in pseudocolor, D). Resting [Ca^2+^]_i _was generally low prior to stimulation (A). Challenging cells for 30 s with 100 μM ATP in presence of extracellular calcium (Ca^2+^_o_) elicited sizeable, albeit variable peak [Ca^2+^]_i _responses from a large pool of chromaffin cells (B). It is noteworthy that some cells either did not respond to ATP or displayed faint responses. These were, for the most part, fully responsive to DMPP or nicotine. It should be emphasized that the concentration of ATP used to stimulate the cells throughout this work (100 μM) is one order of magnitude higher than the minimal concentration necessary to elicit maximal [Ca^2+^]_i _responses, *i.e. *10 μM [7].

There was no apparent differentiation amongst chromaffin cell subtypes with respect to ATP responsiveness in presence of Ca^2+^_o_. Representative time courses of [Ca^2+^]_i _changes for selected chromaffin cells are depicted in Fig. 2E, showing that ATP evoked fast [Ca^2+^]_i _rises followed by decay toward a plateau regardless of whether the cells were adrenergic (e.g. cells 1 and 2) or noradrenergic (e.g. cells 3 and 4).

Cells were perifused with EGTA-containing solutions ([Ca^2+^]_o _~100 nM [7,23]) and subjected shortly after to similar ATP pulses (middle traces in Fig. 2E, peak [Ca^2+^]_i _imaging in Fig. 2C). This protocol, which was designed to minimize calcium depletion of intracellular stores [7,23]), was found in this study to cause extensive depletion in a residual fraction of cells only (see the [Ca^2+^]_i_/UTP experiments below). Cells either did not respond to ATP in the virtual absence of Ca^2+^_o _(e.g. cells 2 and 4) or displayed [Ca^2+^]_i _responses consisting of a rapid rise followed by a decay toward baseline (e.g. cells 1 and 3). This all-or-none response pattern appeared to be unrelated to the specific chromaffin cell phenotype. Cell 5 represents a chromaffin cell that failed to respond to ATP both in high and low Ca^2+^_o_.

Pooling the entire data from Fig. 2 and similar experiments showed that 66% of the whole chromaffin cell population examined (i.e. 392 in 590 cells) responded to ATP in presence of Ca^2+^_o_. Thus, 34% of the cells either lacked Ca^2+^-coupled ATP receptors or had non-functional ATP receptors. Forty two % of the cells displaying positive responses in presence of Ca^2+^_o _(163 in 392) lacked purinergic responses in the virtual absence of Ca^2+^_o_, suggesting that these cells expressed P2X purinergic receptors and did not express Ca^2+^-mobilizing P2Y receptors. Therefore, fifty eight % of the cells displaying positive responses in presence of Ca^2+^_o _were provided with Ca^2+^-mobilizing P2Y purinoceptors, as indicated by the proportion of cells displaying above-threshold ATP-evoked [Ca^2+^]_i _responses in the absence of Ca^2+^_o_. The adopted detection threshold level was a ΔR change of 0.2 (close to the maximal amplitude of the background noise or spurious fluctuations observed under basal conditions).

**Figure 3:**
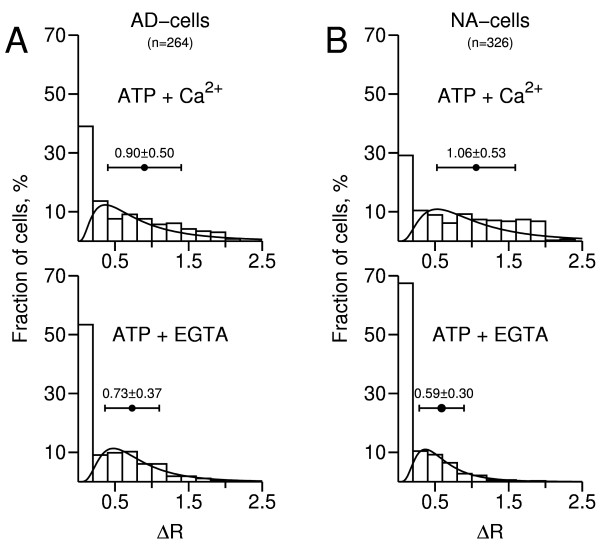
**Frequency distribution histograms of calcium responses to ATP**. Changes in ΔR= F_340_/F_380 _were determined from the experiment depicted in Fig. 2 and 9 similar experiments (n = 590 cells). The first column in each histogram represents unresponsive cells (ΔR < 0.2). "ATP + Ca^2+^": responses obtained in presence of extracellular calcium; "ATP + EGTA": responses obtained in the virtual absence of extracellular calcium. **A**, leftmost histograms: AD-cells; **B**, rightmost histograms: NA-cells. A lognormal distribution function was fitted to each histogram, not taking into account unresponsive cells. The horizontal bars represent mean ΔR ± S.D.

Phenotype-specific analysis of the data is provided in Fig. 3 in the form of frequency distribution histograms of peak ΔR responses to ATP. Both the AD- and NA-subpopulations contained a significant fraction of cells that either lacked Ca^2+^-coupled ATP receptors or had non-functional ATP receptors (39% and 29%, respectively), as depicted by the leftmost columns in the upper histograms. Twenty four % of AD-cells displaying positive responses in presence of Ca^2+^_o _(38 in 161) lacked purinergic responses in the virtual absence of Ca^2+^_o_; the respective figure for NA-cells was 54% (125 in 231 cells). Thus, the fraction of cells that expressed P2X receptors but did not express Ca^2+^-mobilizing P2Y receptors was more than the double in NA-cells relative to the AD-cell subpopulation. In turn, 76% of AD-cells displaying positive responses in presence of Ca^2+^_o _(123 in 161 cells) also responded in the virtual absence of Ca^2+^_o_; the respective figure for NA-cells was 46% (106 in 231 cells). Thus, the fraction of cells expressing Ca^2+^-mobilizing P2Y receptors was approximately 39% higher in the AD-subpopulation. Hence, although expression of P2X and Ca^2+^-mobilizing P2Y receptors cannot be assigned to specific chromaffin cell phenotypes, there appears to be an asymmetric distribution of these receptors amongst AD- and NA-cells, with the latter expressing significantly more P2X and less Ca^2+^-mobilizing P2Y receptors.

As also shown in Fig. 3 (upper histograms), the mean size of the peak ATP-evoked [Ca^2+^]_i _responses, recorded in presence of Ca^2+^_o_, was significantly higher for NA-cells compared to AD-cells (1.06 ± 0.53 vs. 0.90 ± 0.50, n = 161/231 cells; p < 0.005). Since these are probably mixed responses, reflecting activation of both P2 receptor types, the difference might be caused by a higher average density of P2X receptors, Ca^2+^-mobilizing P2Y receptors or both in NA-cells. Indeed, mean ΔR in presence of Ca^2+^_o _was also significantly higher for NA-cells that did not respond to ATP in the virtual absence of Ca^2+^_o _(1.02 ± 0.53 vs. 0.75 ± 0.44 for AD-cells, n = 38/125 cells; p < 0.005). This suggests that individual NA-cells might, on average, have a higher density of P2X receptors. With respect to P2Y receptors, the best possible approach is to compare the ΔR value distributions in the virtual absence of Ca^2+^_o _(lower histograms). Mean ΔR was 0.73 ± 0.37 (n = 123 cells) and 0.59 ± 0.30 (n = 106 cells) for AD- and NA-cells, respectively (significantly different, p < 0.005). This suggests that, on average and contrary to P2X receptors, individual AD-cells might have a higher density of Ca^2+^-mobilizing P2Y receptors.

**Figure 4:**
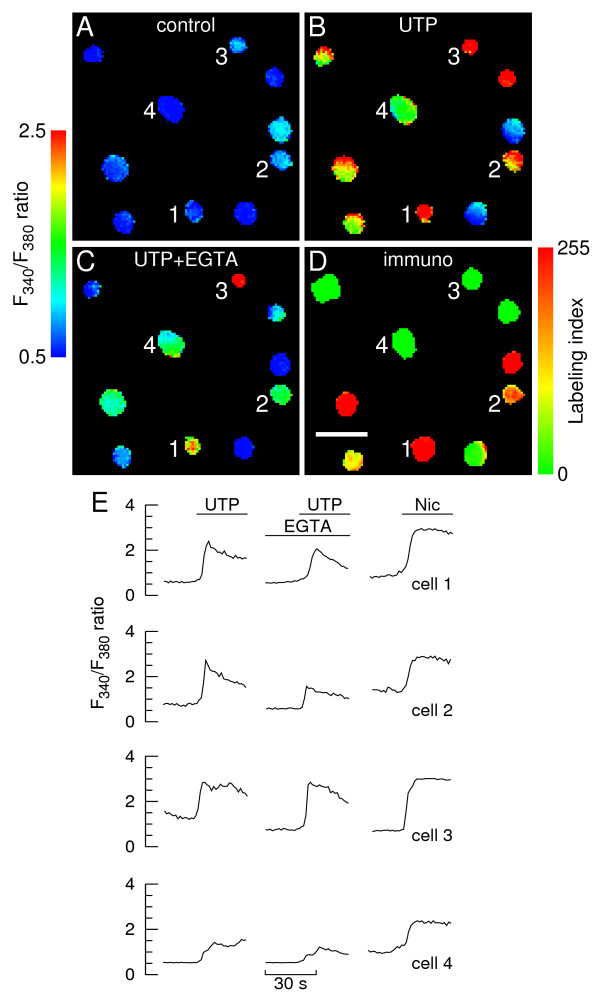
**Calcium responses to UTP in immunolabeled chromaffin cells**. **A-C**. Calcium images showing a group of chromaffin cells before (A, control), during stimulation with 100 μM UTP in presence of extracellular calcium (B) and during stimulation in the virtual absence of extracellular calcium (C, "UTP+EGTA"). At the end of each experiment cells were stimulated with 10 μM nicotine. Cells were allowed to rest for 10 min between consecutive stimulations. The fura-2 fluorescence ratio F_340_/F_380 _was determined for each cell in a field on a pixel-by-pixel basis. Images were coded in pseudocolor to show differences in the F_340_/F_380 _ratio. The images corresponding to UTP stimulation (B and C) were captured ~5 s after the UTP challenges (i.e. at the response peak in presence of extracellular calcium); **D**. Pseudocolor image of PNMT/TH immunofluorescence ratio (labeling index) and corresponding scale. Intensely colored red and green cells are adrenergic and noradrenergic cells, respectively. Scale bar = 50 μm; **E**. Time courses of changes in F_340_/F_380 _fluorescence ratio for two AD-cells (cells 1 and 2, also depicted in A-D) and two NA-cells (cells 3 and 4). The lines denote superfusions with UTP or nicotine in the presence or virtual absence (middle traces) of extracellular calcium.

The following experiments were designed to assess the [Ca^2+^]_i _responses of immunolabeled chromaffin cells to activation of UTP-sensitive receptors (henceforth designated "P2U receptors" for simplicity). Typical pseudocolor fura-2 fluorescence images are depicted in Fig. 4(A–C) , together with the respective immunostaining identification (D) and representative time courses of [Ca^2+^]_i _changes for selected chromaffin cells (E). As for ATP, applying brief pulses of 100 μM UTP in presence of Ca^2+^_o _elicited sizeable, albeit variable peak [Ca^2+^]_i _responses from both AD-cells (e.g. cells 1 and 2) and NA-cells (e.g. cells 3 and 4) (B and E). The predominant response pattern was a fast rise, followed by a slow decay which did not reach baseline by the end of the 30 s UTP challenge. The majority of cells displaying positive responses in presence of Ca^2+^_o _yielded sizeable, albeit somewhat diminished responses to the purinergic agonist in the virtual absence of Ca^2+^_o_, regardless of the respective phenotype. It is noteworthy that, under these conditions, the [Ca^2+^]_i _tended to decay toward baseline at a faster rate throughout agonist exposure. A few cells did not respond to UTP in the virtual absence of Ca^2+^_o _while yielding a sizeable response in its presence. It should be emphasized that the concentration of UTP used to stimulate the cells throughout this work (100 μM) evokes maximal [Ca^2+^]_i _responses from bovine chromaffin cells [8].

Pooling the entire data from Fig. 4 and similar experiments showed that 63% of the whole chromaffin cell population examined (i.e. 182 in 288 cells) responded to UTP in presence of Ca^2+^_o_, indicating that these cells expressed functional "P2U receptors". This figure is close to the fraction of chromaffin cells (66%) that have been shown to express functional ATP receptors in the former experiments where ATP was used as a purinergic agonist (Figs. 2 and 3). This was an unexpected finding, since UTP (admittedly a purinergic agonist specific for selected P2Y receptor subtypes) should probe a subset of the ATP-sensitive receptor subtypes. There are several possibilities to account for this apparent discrepancy. First, UTP might have ionotropic-like effects in chromaffin cells. Secondly, the integrity of P2X and/or P2Y receptors might be highly sensitive to the harshness of collagenase digestion (and therefore prone to undergo spurious changes from batch to batch). Finally, the cell pool used in the [Ca^2+^]_i_/UTP experiments might contain an exceedingly higher proportion of AD-cells (which express more P2Y receptors than NA-cells, see above). This proportion was indeed 59% (*vs. *45% in the former [Ca^2+^]_i_/ATP experiments). The "UTP specificity" hypothesis is ruled out by the intracellular sodium experiments (see below).

**Figure 5:**
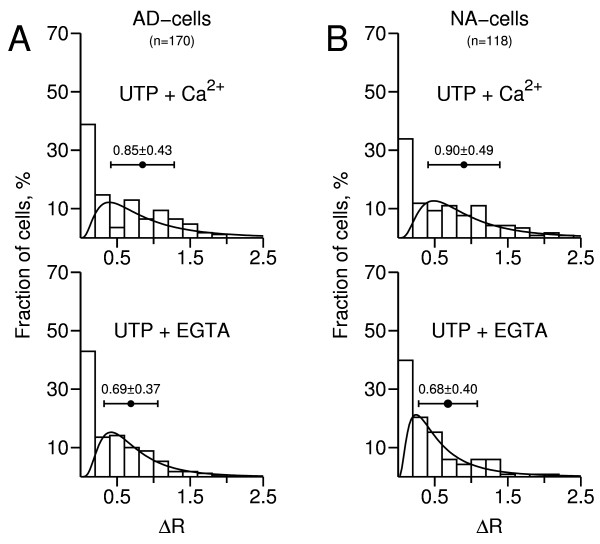
**Frequency distribution histograms of calcium responses to UTP**. Changes in ΔR = F_340_/F_380 _were determined from the experiment depicted in Fig. 4 and 5 similar experiments (n = 288 cells). The first column in each histogram represents unresponsive cells (ΔR < 0.2). "UTP + Ca^2+^": responses obtained in presence of extracellular calcium; "UTP + EGTA": responses obtained in the virtual absence of extracellular calcium. **A**, leftmost histograms: AD-cells; **B**, rightmost histograms: NA-cells. A lognormal distribution function was fitted to each histogram, not taking into account unresponsive cells. The horizontal bars represent mean ΔR ± S.D.

Phenotype-specific analysis of the data using frequency distribution histograms (Fig. 5) shows that both the AD- and NA-subpopulations contained a significant fraction of cells that either lacked or had non-functional "P2U receptors" (38% and 34%, respectively), as depicted by the leftmost columns in the upper histograms.

Moreover, 93% of the AD-cells (97 in 104) displaying positive UTP responses in presence of Ca^2+^_o _also displayed detectable responses in the virtual absence of Ca^2+^_o_; the respective figure for NA-cells was 91% (71 in 78 cells). This indicates that subjecting the vast majority of cells to EGTA-containing solutions did not cause extensive depletion of internal Ca^2+ ^stores.

As also shown in Fig. 5 (upper histograms), the mean size of the UTP-evoked [Ca^2+^]_i _responses, recorded in presence of Ca^2+^_o_, was not significantly different between AD- and NA-cells (0.85 ± 0.43 *vs*. 0.90 ± 0.49, n = 118/170 cells; p = 0.5). Nor was the mean size recorded in the virtual absence of Ca^2+^_o _(0.69 ± 0.37 and 0.68 ± 0.40 for AD- and NA-cells, respectively; n = 71/97 cells, p = 0.9; lower histograms). This suggests that, in contrast to the situation found for Ca^2+ ^mobilizing P2Y receptors in the [Ca^2+^]_i_/ATP experiments, individual AD- and NA-cells might have a similar average density of "P2U receptors".

Subjecting AD-cells to EGTA-containing solutions reduced significantly (19%) the mean size of the UTP-evoked [Ca^2+^]_i _responses (0.69 ± 0.37 *vs*. 0.85 ± 0.43 for high Ca^2+^_o_; n = 97 cells, p < 0.005). A similar observation was made for NA-cells (0.68 ± 0.40 *vs*. 0.90 ± 0.49 for high Ca^2+^_o_, a 24% change; n = 71/118 cells, p < 0.0001). The simplest explanation for these differences is that reducing extracellular free Ca^2+ ^to around 100 nM depleted intracellular Ca^2+ ^stores by approximately 20% in both cell types.

### [Na^+^]_i _responses to purinergic agonists in chromaffin cell subtypes

The following experiments were designed to investigate the specificity of UTP as a P2Y receptor agonist in chromaffin cells, as well as to further assess the possibility that P2X receptors might be differentially distributed among AD- and NA-cells. When activated, these receptors allow for pronounced Na^+ ^influx and concomitant increases in cytosolic free Na^+ ^concentration ([Na^+^]_i_) [35], which in the present work were assessed by sodium-binding benzofuran isophthalate (SBFI) fluorescence imaging. The adopted detection threshold level was a ΔR change of 0.05.

**Figure 6:**
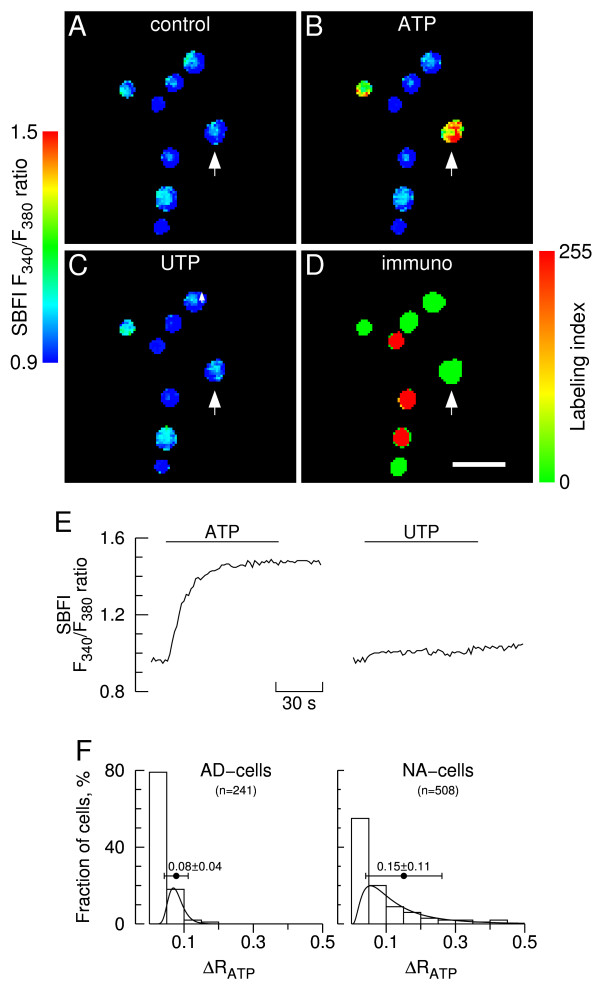
**Sodium responses to ATP and UTP in immunolabeled chromaffin cells**. **A-C**. Sodium images showing a group of chromaffin cells during stimulation with 100 μM ATP (B) and UTP (C) in presence of extracellular calcium. Panel A depicts cells prior to stimulation (control). Cells were allowed to rest for 30 min between consecutive stimulations. The SBFI fluorescence ratio F_340_/F_380 _was determined for each cell in a field on a pixel-by-pixel basis. Images were coded in pseudocolor to show differences in the F_340_/F_380 _ratio. The images corresponding to ATP and UTP stimulation (B and C) were captured ~30 s after challenges (response peaks); **D**. Pseudocolor image of PNMT/TH immunofluorescence ratio (labeling index) and corresponding scale. Intensely colored red and green cells are adrenergic and noradrenergic cells, respectively. Scale bar = 50 μm. **E**. Time course of changes in F_340_/F_380 _fluorescence ratio for an NA-cell (depicted by an arrow in A-D). The lines denote superfusions with ATP and UTP; **F**. Frequency distribution histograms of sodium responses to ATP. Changes in ΔR= F_340_/F_380 _were determined from the experiment depicted in A-B and 11 similar experiments (n = 749 cells). The first column in each histogram represents ATP-unresponsive cells (ΔR < 0.05). Leftmost histogram: AD-cells; rightmost histogram: NA-cells. A lognormal distribution function was fitted to each histogram, not taking into account unresponsive cells. The horizontal bars represent mean ΔR ± S.D.

Pseudocolor SBFI fluorescence images are depicted in Fig. 6(A–C), together with the respective immunostaining identification (D) and a representative time course of [Na^+^]_i _changes (E). ATP evoked rapid and sustained [Na^+^]_i _rises in a fraction of the cells (B and E), followed by a slow return toward basal levels (not depicted in the figure). Subsequent application of UTP failed to raise the [Na^+^]_i _in all cells tested (n = 749, C and E). Thus, activation of "P2U receptors" is not coupled to Na^+ ^influx and UTP does not activate P2X receptors.

Only a fraction of the cells (21% AD-cells and 45% NA-cells) displayed positive responses to ATP ([Na^+^]_i _rises) according to the detection threshold level adopted; the remainder failed to respond, as depicted by the leftmost columns of the frequency distribution histograms (Fig. 6F). Moreover, the mean size of the ATP-evoked [Na^+^]_i _responses was significantly higher for NA-cells compared to AD-cells (0.15 ± 0.11 vs. 0.08 ± 0.04, n = 51/231 cells; p < 0.0001). It is noteworthy that the mean size of the ATP-evoked [Na^+^]_i _responses in AD-cells was just slightly above the detection threshold level. This reinforces the view that NA-cells might have a higher average density of P2X receptors.

## Discussion

A significant fraction of the chromaffin cell population used in this work (~30%) did not respond to ATP in presence of extracellular Ca^2+^, suggesting that these cells either lacked Ca^2+^-coupled purinergic receptors or that the existing receptors were rendered non-functional owing to enzymatic treatment. As revealed by the [Ca^2+^]_i_/ATP experiments, approximately 40% of the cells displaying functional purinoceptors expressed P2X receptors but did not express Ca^2+^-mobilizing P2Y receptors; approximately 60% yielded positive responses to ATP in the virtual absence of extracellular Ca^2+^, indicating that they expressed Ca^2+^-mobilizing P2Y receptors. Coexistence of P2X and P2Y receptors in a limited fraction of the latter cells cannot be ruled out, as suggested by an earlier study from our laboratory [23].

As revealed by the [Ca^2+^]_i_/ATP and [Na^+^]_i _experiments (which accounted for over 85% of the cells examined), relative to AD-cells more than the double of individual NA-cells expressed P2X receptors and did not express Ca^2+^-mobilizing P2Y receptors. Moreover, the density of P2X receptors in NA-cells appeared to be 30–50% larger, as suggested by comparing the average size of the [Ca^2+^]_i _and [Na^+^]_i _responses. Thus, although P2X receptors cannot be ascribed to a specific cell phenotype they are preferentially located to the NA-cell subpopulation. Conversely, by the same criteria approximately twice as many AD-cells expressed Ca^2+^-mobilizing P2Y receptors, and they appeared to exhibit a higher (~20%) receptor density. Hence, the distribution of Ca^2+^-mobilizing P2Y receptors is also asymmetric, with these receptors being preferentially located to the AD-cell subpopulation.

The cell pool used for the [Ca^2+^]_i_/UTP experiments (~15% of the cells examined in this study) displayed a disproportionately high fraction of cells expressing Ca^2+^-mobilizing P2Y receptors. This cannot be attributed to lack of specificity of UTP as a P2Y receptor agonist, as demonstrated by the [Na^+^]_i _experiments (UTP did not evoke Na^+ ^influx in any of the cells tested). A possible explanation is that, compared to the [Ca^2+^]_i_/ATP experiments, the cell pool was somewhat enriched in AD-cells. Another plausible explanation is that P2X receptors might be more prone to damage during the collagenase-isolation procedure, thereby causing an artifactual and sporadic enrichment of functional P2Y receptors. It is noteworthy in this respect that, in several studies reporting an electrophysiological analysis of the modulatory effect of P2Y receptor activation on voltage-sensitive Ca^2+ ^currents and exocytosis, extracellular ATP either failed to evoke inward currents and secretion or these effects were observed sporadically [4,9-14] (but see others where clear-cut effects of P2X agonists have been observed [31,32]). Nonetheless, the [Ca^2+^]_i_/UTP experiments were instrumental to assess the possibility that reducing extracellular Ca^2+ ^to ~100 nM might cause extensive depletion of intracellular Ca^2+ ^stores. This was not the case, as indicated by the residual number of cells that lost ATP responsiveness when exposed to EGTA-containing solutions.

Interestingly, the density of UTP-sensitive P2Y receptors did not vary with the chromaffin cell phenotype, as suggested by comparing the average size of the [Ca^2+^]_i _responses between AD- and NA-cells. Taking into account the homologous data from the [Ca^2+^]_i_/ATP experiments, which hinted at a higher Ca^2+^-mobilizing P2Y receptor density in AD-cells (see above), this suggests that ATP might, in addition to the "P2U receptor", activate an UTP-insensitive Ca^2+^-mobilizing P2Y receptor subtype in bovine chromaffin cells (see [33] for further evidence). This is not unlikely, as distinct P2Y subtypes coexist in neurons and other cells [24,26,36]. One possibility is the P2Y_12 _receptor reported by Ennion *et al. *[13], which however did not appear to be coupled to Ca^2+ ^release from intracellular stores, at least under the stringent experimental conditions imposed by the authors. It is also noteworthy that Ennion *et al. *detected transcripts for P2Y_1 _receptors by RT-PCR analysis. Work is now in progress to identify the putative UTP-insensitive P2Y receptor.

The present finding that P2X receptors are preferentially expressed in noradrenergic bovine chromaffin cells agrees with earlier observations that ATP evokes the preferential, if not exclusive release of norepinephrine from chromaffin cell preparations [6,23]. There is evidence that stimulated Ca^2+ ^influx is strongly coupled to catecholamine release; in contrast, Ca^2+ ^release from intracellular stores (a characteristic feature of most G_i/o_- and G_q/11_-coupled P2Y receptor subtypes [24]) appears to be loosely coupled to secretion [15,23]. Activation of P2Y receptors is actually known to inhibit evoked neurotransmitter release, notably from sympathetic and noradrenergic brain neurons [37]. The same holds for chromaffin cells, where P2Y receptor activation inhibits voltage-sensitive Ca^2+ ^channels via G_i_/G_o _proteins and, thus, depresses Ca^2+^-dependent exocytosis [4,9-14]. Activation of P2Y receptors also inhibits catecholamine secretion downstream Ca^2+ ^influx in rat chromaffin cells [38]. Although the role for these modulations remains unclear, it has been suggested that they might be an essential component of an auto-inhibitory loop involving granule-stored ATP. Besides ATP other endogenous modulators (e.g. opioid peptides and catecholamines) have been involved in autocrine/paracrine interactions within the adrenal medulla [39-42].

Excessive release of epinephrine following an acute stress episode may cause irreversible damage of the myocardium and other dysfunctions, eventually leading to death. Not considering auto-inhibitory feedback loops related to cholinergic transmission, inhibitory transmitters released from nerve terminals within the adrenal medulla (e.g. opioid peptides, ATP and norepinephrine), inhibitory transmitters or mediators released from chromaffin cells (e.g. opioid peptides, norepinephrine, ATP and chromogranin A-derived fragments) or molecules arising from the degradation of any of these (e.g. adenosine *via *ectonucleotidases) are well suited to prevent that from occurring provided that the respective receptors are preferentially located at adrenergic chromaffin cells. The available evidence indicates that this likely is the case for ATP and opioid peptides. Indeed, the adrenergic cell subpopulation is enriched in P2Y (this work) and κ-opioid receptors [21]. Furthermore, endogenous agonists for both receptors exert profound inhibitory actions on voltage-sensitive Ca^2+ ^channels and exocytosis [4,9,10,12-14,40-44]. There is also compelling evidence for short-loop feedback inhibition of epinephrine release by norepinephrine (*via *α_2C_-adrenoceptors) [45].

**Figure 7:**
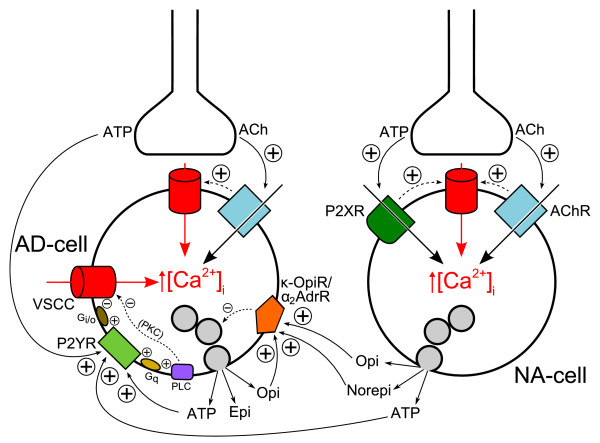
**Symplified model for inhibitory regulation of epinephrine secretion involving transmitters released from both nerve terminals and chromaffin cells**. Auto-inhibitory feedback loops related to cholinergic transmission are not considered for simplicity. Inhibitory transmitters acting on receptors preferentially located to adrenergic chromaffin cells (i.e. P2Y receptors (this work) and κ-opioid receptors [21]) have been considered, as well as norepinephrine which inhibits epinephrine release *via *α_2C_-adrenoceptors [45]. Activation of P2Y, κ-opioid and α_2_-adrenergic receptors inhibits voltage-sensitive Ca^2+ ^channels *via *G_i/o _proteins (not depicted for the latter two receptors for simplicity) and, consequently, exocytosis [4,9,10,12-14,40-45]. Protein kinase C is negatively coupled to VSCCs in an isoform-specific fashion [46]. AD-cell: adrenergic chromaffin cell; NA-cell: noradrenergic chromaffin cell; Ach: acetylcholine; VSCC: voltage-sensitive Ca^2+ ^channels; AchR: nicotinic cholinergic receptors; P2XR: P2X receptors; P2YR: P2Y receptors; κ-OpiR/α_2_AdrR: κ-opioid and α_2_-adrenergic receptors (represented as a single entity for simplicity); PLC: phospholipase C; PKC: protein kinase C; G_q _and G_i/o_: G proteins; Epi: epinephrine; Norepi: norepinephrine; Opi: opioid peptides. For simplicity, and because [Ca^2+^]_i _rises induced by PLC activation do not evoke catecholamine secretion from bovine chromaffin cells, they are not made explicit in the scheme. Also for simplicity, granule exocytosis is not depicted as occurring preferentially in the vicinity of VSCC hot-spots. Positive and negative signs indicate stimulatory and inhibitory interactions, respectively.

In the light of the present results, we propose a mechanism (Fig. 7) whereby: 1) ATP is initially co-released from cholinergic nerve endings, facilitating norepinephrine release from NA-cells *via *fast P2X receptor-coupled channels; 2) ATP, co-released with both epinephrine and norepinephrine, acts upon P2Y receptors in AD-cells, inhibiting Ca^2+ ^influx and contributing to terminate epinephrine secretion after a diffusion delay. This inhibition may be effected on voltage-sensitive Ca^2+ ^channels directly by G_i_/G_o _proteins or indirectly by specific PKC isoforms, as demonstrated by our laboratory [46]. (As noted above, activation of P2Y receptors elicits Ca^2+ ^release from intracellular stores which, however, does not evoke catecholamine secretion from bovine chromaffin cells. Secretion appears to occur from discrete exocytotic sites [47]. Thus, the focal Ca^2+ ^signal may dissipate to the extent that these sites are not activated. Ca^2+ ^release from intracellular stores may nonetheless be relevant to modulate other cellular targets, including the nucleus.) Short-loop feedback inhibition of epinephrine release by activation of κ-opioid and α_2_-adrenergic receptors is also depicted in the model (Fig. 7). Finally, adenosine arising from the degradation of extracellular ATP may contribute to the inhibitory loop [48].

## Conclusion

Although neither of the major purinoceptor types can be ascribed to a particular cell phenotype, P2X and Ca^2+^-mobilizing P2Y receptors are preferentially located to noradrenergic and adrenergic chromaffin cells, respectively. ATP might, in addition to an UTP-sensitive P2Y receptor, activate an UTP-insensitive P2Y receptor subtype. A model for a short-loop feedback interaction is presented whereby locally released ATP acts upon P2Y receptors in adrenergic cells, inhibiting Ca^2+ ^influx and contributing to terminate epinephrine secretion evoked by splanchnic nerve stimulation.

## Methods

### Cell culture

Bovine adrenal glands were obtained from the local slaughterhouse and kept on ice during transportation. Adrenal medulla cells were isolated by collagenase digestion of the glands and purified on a Percoll density gradient essentially as described previously [49,50]. The purified cell fraction thus obtained is enriched in chromaffin cells. Cells were cultured under a 5% CO_2_/95% air humidified atmosphere in a 1:1 mixture of Dulbecco's modified Eagle's medium (DMEM)/Ham's F-12 medium buffered with 15 mM N-2-hydroxyethylpiperazine-N'-2-ethanesulphonic acid (HEPES) and 25 mM NaHCO_3_, supplemented with 5% heat-inactivated fetal calf serum, penicillin (100 units/ml) and streptomycin (100 μg/ml) (Biological Industries, Beth Haemek, Israel). The cells were plated on grid-etched glass coverslips coated with poly-L-lysine. Cells were typically used between days 2 and 5 after plating.

### Solutions

The Ca^2+^-containing salt solution used in the imaging experiments had the following composition (mM): 140 NaCl, 5 KCl, 2 CaCl_2_, 1 MgCl_2_, 10 mM HEPES and 10 glucose (pH 7.4). In some experiments extracellular free [Ca^2+^] was buffered at 100 nM by mixing appropriate amounts of Ca^2+ ^and EGTA, as described elsewhere [51].

### [Ca^2+^]_i _and [Na^+^]_i _imaging

The coverslips containing the cells were washed in physiological saline supplemented with 1% bovine serum albumin (BSA). The cells were then loaded with either 2.5 μM fura-2/AM (the acetoxymethyl ester of fura-2, [Ca^2+^]_i _measurements [52]) or 10 μM SBFI ([Na^+^]_i _measurements [53]) for 45 min at 37°C in this medium, under a 95% O_2_/5% CO_2 _atmosphere. After loading, the coverslips were washed and maintained in BSA-containing solution at room temperature. The loaded cells were assayed within 90 min from the end of loading. Each coverslip was glued to the bottom of a small (approximately 100 μl) perifusion chamber and placed on the stage of a Nikon Diaphot inverted fluorescence microscope. The cells were continuously perifused (approximately 1.5 ml/min) with physiological saline at room temperature. The solution was fed into a four-way stopcock valve located near the recording chamber. The fluorescence changes were recorded using a multiple excitation MagiCal imaging system (Applied Imaging, U.K.), essentially as described [17]. Briefly, chromaffin cells were alternately excited at 340 and 380 nm by means of a stepping filter wheel and the epifluorescence optics of the microscope. Emitted fluorescence collected with a 20× objective was driven to a Photonics Science SIT camera after passing through a 510 nm bandpass filter. Eight frames (approximately 100 ms exposure) were averaged to produce each image. Alternating excitation, image capture and processing were controlled by a single processor in the MagiCal system. Image analysis was performed with in-house and commercially available software. Essentially, background fluorescence at each wavelength (obtained from a field devoid of cells in each coverslip) was subtracted and fluorescence images ratioed on a pixel-by-pixel basis. Ratio data were stored as 8-bit pseudocolored images. A contour was drawn around each cell in a field and the averaged ratio value of pixels inside each contour evaluated at each time point, in order to obtain ratio *vs. *time plots for all cells.

### Immunocytochemical identification

Following the microfluorescence experiments the cells attached to the coverslips were fixed for 2 min in ice-cold acetone/methanol (1:1, v/v), and stored frozen (-20°C) until further processing. For immunolabeling the coverslips were thawed in acetone/methanol, washed in phosphate buffer saline (PBS) and blocked for 1 h with PBS containing 3% BSA, 1% normal goat serum (NGS) and 0.1% Triton X-100. The following primary antibodies were used for specific labeling of adrenergic chromaffin cells and all adrenergic + noradrenergic chromaffin cells: polyclonal rabbit anti-PNMT (1:1000, Affinity Research, Exeter, Devon, UK) and monoclonal mouse anti-TH (1:100, Boehringer Mannhein, Germany), respectively. Cells were incubated with primary antibodies for 1 h at 37°C, and washed in PBS containing 0.1% Triton X-100. Fluorescence labeling of AD-cells and the whole chromaffin cell population was carried out by incubating the cells for 1 h with the following secondary antibodies: TRITC (tetramethylrhodamine isothiocyanate)-conjugated anti-rabbit IgG and FITC (fluorescein isothiocyanate)-conjugated anti-mouse IgG at 1:200 (Sigma, St. Louis, MO, USA), respectively.

Immunocytochemical controls were prepared as described, omitting the primary antibodies and incubating the cells with 3% BSA instead. Thus, these controls assessed nonspecific binding of labeled secondary antibodies to sample structures. Immunofluorescence images were acquired by the MagiCal imaging system, using appropriate fluorescein and rhodamine filters (Omega Optical, Brattleboro, VT, USA). A quantitative labeling index (rhodamine/fluorescein fluorescence intensity ratio) was established to identify chromaffin cell subtypes (see above). As for the intracellular calcium and sodium experiments, ratioing immunofuorescence intensities was expected to cancel errors arising from heterogeneous illumination and other instrumental factors. The coverslips used in the experiments were grid-etched, so that single cell calcium and sodium responses could be assigned to particular chromaffin cells.

### Statistical analysis

Data are presented as mean ± S.D. Statistical significance of differences was assessed by paired (within the same experiment) or unpaired (between experiments) Student's t-test; differences were considered significant at the 95% confidence level (P < 0.05). A gamma distribution function was used to fit the histogram in Fig. 1 (not shown); a lognormal distribution function was used to fit the skewed distribution histograms in Figs. 3, 5 and 6, skipping the data relative to the first bin (ΔR < 0.2 for Figs. 3 and 5 or ΔR < 0.05 for Fig. 6). Fitting the data to probability density functions was made with gnuplot [54] following the guidelines of the Statistical Engineering Division of the National Institute of Standards and Technology [55].

### Other materials

Fura-2/AM and SBFI were from Molecular Probes (Eugene, Ore., USA). ATP was from Boehringer (Mannheim, Germany). Unless otherwise specified, all other chemicals were from Sigma Chemical Co. (St. Louis, Mo., USA).

## Authors' contributions

ART carried out the experiments, performed the statistical analysis, prepared the figures, participated in conceiving and designing the study, and helped to draft the manuscript; EC helped in carrying out the experiments and participated in conceiving and designing the study; RMS helped to draft the manuscript; LMR participated in conceiving and coordinating the study and drafted the manuscript. All authors read and approved the final manuscript.
